# Food ingredients and food made with plant cell and tissue cultures: State‐of‐the art and future trends

**DOI:** 10.1002/elsc.202000077

**Published:** 2021-01-19

**Authors:** Geraldine Gubser, Sabine Vollenweider, Dieter Eibl, Regine Eibl

**Affiliations:** ^1^ Institute of Chemistry and Biotechnology Zurich University of Applied Sciences (ZHAW) Wadenswil Switzerland; ^2^ Natural Ingredient Development (NID), Givaudan Kemptthal Switzerland

**Keywords:** adventitious root culture, elicitation, food colorants, health food ingredients, suspension culture

## Abstract

Climate change and an increasing world population means traditional farming methods may not be able to meet the anticipated growth in food demands. Therefore, alternative agricultural strategies should be considered. Here, plant cell and tissue cultures (PCTCs) may present a possible solution, as they allow for controlled, closed and sustainable manufacturing of extracts which have been or are still being used as colorants or health food ingredients today. In this review we would like to highlight developments and the latest trends concerning commercial PCTC extracts and their use as food ingredients or even as food. The commercialization of PCTC‐derived products, however, requires not only regulatory approval, but also outstanding product properties or/and a high product titer. If these challenges can be met, PCTCs will become increasingly important for the food sector in coming years.

**Abbreviations**
2.4‐D: 2,4‐dichlorophenoxyacetic acid
ABR: Active Botanicals Research
CMC: Cambial Meristematic Cell
D: Dark
DDC: Dedifferentiated Cell
EFSA: European Food Safety Authority
EU: European Union
FDA: Food and Drug Administration
GRAS: Generally Recognized As Safe
IRB: Instituto di Ricerche Biotecnologiche S.R.l.
L: Light
PCTC: Plant Cell and Tissue Culture
VTT: Technical Research Center of Finland Ltd.

## 1 INTRODUCTION

There are currently more than 7 billion people on earth, with predictions by the United Nations estimating that this number is set to increase to 10 billion by 2050 [1]. While this has led to increased demands for food, the limited amount of arable land per capita available for food production has constantly been decreasing due to population growth and factors such as urbanization, erosion, soil salinization, and desertification. Furthermore, sustainability in food production [2, 3] and the threat of crop losses due to climate change and plant diseases are playing an increasingly important role and need to be taken into account. These factors could be countered using new and ethically more justifiable technologies such as cellular agriculture, which aims to produce agricultural products such as textiles, leather, meat, milk or egg proteins or fats without plants or animals in bioreactors, utilizing the cells of microorganisms, animals or plants as renewable factories. The products produced in this manner are either cellular or acellular in nature [1, 2] and while the commercially available acellular products are usually produced with genetically modified organisms, their cellular counterparts are still based on native organisms [3].

Commercial products based on plant cell and tissue cultures (i.e. aseptically isolated plant tissues and cells cultivated under defined chemical and physical conditions) have been on the market since the 1980s [4]. They represent ingredients for products of the pharmaceutical, cosmetics and food industries. These products, the majority of which are cosmetic products, can be produced in a controlled manner under closed conditions independent of climate, weather and soil conditions. Variations in product quantity and quality, which are typical for the use of collected or cultivated whole plants, can be avoided [5, 6]. In addition, the use of protected and endangered plant species becomes possible and the metabolism of the established plant cell and tissue culture (PCTC) can be specifically influenced. In other words, the formation of substances that are beneficial to the consumer can be supported and those that are harmful to the consumer can be reduced or even suppressed.

Starting with an overview of commercial PCTC‐based food ingredients, this review intends to shed light on select details, application principles, as well as technologies used for the manufacture of PCTC‐based food and food ingredients, including approaches to increasing process efficiency. In addition, the latest developments aimed at producing new PCTC extracts with nutritional potential and PCTC‐based flavour production is briefly discussed. Finally, the authors address regulatory issues regarding the application of PCTC‐based products in the food industry.

## 2 COMMERCIAL FOOD INGREDIENTS BASED ON PLANT CELL CULTURE TECHNOLOGY

### 2.1 Product overview

In Table 1 PCTC‐based food ingredients which have been commercialized at an industrial scale are summarized. The list of products is assigned to two product classes based on their application: (1) colorants and (2) compounds with health promoting effects. The colorants cover anthocyanins, betacyanins and shikonin. Production processes for these colorants run for a maximum of 23 day, with bioreactors using maximum working volumes of between 500 L [7] to 600 L [4, 8, 9] and, excepting the production of betacyanin [10, 11, 12, 13], delivering product yields up to the kilogram range. The commercial production of shikonin, the first authorized secondary metabolite worldwide, with *Lithospermum erythrorhizon* is particularly noteworthy, as the PCTC‐based process of Mitsui Petrochemical Industries Ltd. allows for the production of 5 kg of pure shikonin per bioreactor run. With comparable shikonin quality, productivity is about 800 times higher than with the traditional process using ginseng roots grown in a field [4, 14]. Another remarkable example of a PCTC‐based process is the scale‐up of echinacoside manufacturing to a production bioreactor size of 75 m^3^, which was achieved by the Diversa Gesellschaft für Bio‐ und Verfahrenstechnik in Germany in the 1990s [15]. This company, which came to be known as Phyton Biotech, ceased the production of echinacoside in favor of paclitaxel and has been successfully manufacturing this product in bioreactors for more than 20 years [16, 17].

**TABLE 1:** Overview of commercialized food colorants and health food ingredients based on PCTCs (arranged in alphabetic order)

Classification	Compound	Plant species, cell culture type	Manufacturer	Commercial availability	Reference
Colorant	Anthocyanins	*Euphorbia milii*, suspension culture^e^	Nippon Paint Co. Ltd.	Not clear	[18, 19]
		*Aralia cordata*, suspension culture^e^	Tonen Co. Ltd.	Not clear	[7, 20, 21]
	Betacyanins	*Beta vulgaris *L., suspension culture^e^	Nippon Shinyaku Co. Ltd.	Not clear	[10, 12, 13, 22, 23]
			Somar Corporation	Not clear	[11]
	Shikonin	*Lithospermum erythrorhizon*, suspension culture^e^	Mitsui Petrochemical Industries^a^	Not clear^g^	[4, 8, 9, 14]
Health food ingredient	Echinacosides	*Echinacea angustifolia*, suspension culture^e^ *Echinacea purpurea*, suspension culture^e^ and adventitious root culture	ABR	Product available, novel food (Europe)	[24, 25]
			CBN Plantech Co. Ltd	Not clear	[26]
			IRB	Product available, novel food (Europe)	[27, 28]
			Diversa Gesellschaft für Bio‐ und Verfahrens‐technik mbH^c^	Commercial manufacture stopped in 1993	[15, 16, 17]
	Ginseng saponins	*Panax ginseng*, adventitious root culture	CBN Biotech	Product approved (Korean Food and Drug Association and FDA)	[29, 30, 31, 32, 33, 34]
			Nitto Denko Corporation	Not clear	[35, 36]
		*Wild ginseng*, suspension culture^f^	Unhwa Corporation	Product approved as healthcare supplement	[37, 38]

Classification	Compound	Plant species, cell culture type	Manufacturer	Current status	Reference
Health food ingredients	Cocoa polyphenols	*Theobroma cacao*, suspension culture^e^	Diana Plant Sciences^d^	Commercial manufacture stopped in 2014	[39, 40, 41]
	Teupoloside	*Ajuga reptans*, suspension culture^f^	ABR, IRB^b^	Product available, novel food (Europe)	[24, 42, 43]
	Verbascoside	*Lippia citriodora*, suspension culture^f^	ABR	Product available, novel food (Europe)	[25, 44, 45]

^a^ Now Mitsui Chemicals, Inc.

^b^ Now Part of Croda International.

^c^ Now Phyton Biotech.

^d^ Now part of Symrise AG.

^e^ Dedifferentiated cells (DDCs).

^f^ Cambial meristematic cells (CMCs).

^g^ Preferably used as colorant in the cosmetics industry (products such as lipsticks, lotions and soaps are no longer available).

Active Botanicals Research (ABR) and the Instituto di Ricerche Biotecnologiche S.R.l. (IRB) claim on their website that they produce “Echinan 4P” and “Echigena plus” from cell cultures of *Echinacea angustifolia* in production facilities up to m^3^ scale, however no detailed information on the production process has been published. CBN Plantech Co. Ltd. also works with *Echinacea angustifolia* based cell cultures at a scale of up to 500 L. Following one week of cultivation, 1.75 kg dry biomass containing 33.44  mg g^–1^ total caffeic acid can be harvested [26] of which an accumulation of 12.3 mg g^–1^ echinacoside has been reported.

Another breakthrough was the PCTC‐based production of ginseng saponins, as the traditional agricultural cultivation of ginseng is considered a very time‐consuming and labor‐intensive process, requiring up to 7 years. Nitto Denko Corporation scaled up the cultivation of *Panax ginseng* cell cultures to a 2 m^3^ scale, achieving 19 g L^–1^ dry cell biomass (700 mg L^–1^ d^–1^) in merely 4 weeks, and receiving permission for commercial use of the manufactured biomass as a food additive in Japan in the late 1980s [35]. Further attempts to produce ginseng saponins from PCTCs have also been made by CBN Biotech and Unhwa Corporation.

Through bioreactor and process optimization (see also Sections 2.2.2 and 2.2.3) CBN Biotech was able to achieve a total saponin content (5% of cell dry weight) more than twice as high as that of field‐grown ginseng in 0.5 and 1 m^3^ bioreactors [29]. The 10 m^3^ production bioreactor version provides an average production of 45 t of biomass fresh weight per year [30, 31]. Unhwa Corporation, on the other hand, succeeded in establishing a highly productive biotechnological production process by cultivating cambial meristematic cells (see Section 2.2.1) of wild ginseng for the first time [37, 38]. The product for the food sector, DDB20, has been on the market since 2014. In the same year, the first cocoa PCTC‐derived nutraceutical from the US company Diana Plant Sciences received the Global Frost & Sullivan Award for Visionary Innovation Leadership. It was named Cocovanol and was characterized by a higher flavanol content than basic cocoa, with only traces of caffeine and theobromine [46, 47]. However, since the acquisition of Diana Plant Sciences by Symrise 6 years ago, no information has been made available regarding this product. Finally, it should be mentioned that Active Botanicals Research and the Instituto di Ricerche Biotecnologiche S.R.I., in addition to the PCTC‐based echinacoside‐containing products ECHINAN 4P and ECHIGENA PluS, manufacture the teupoloside products TEUPOL 10P and 50P as well as TEOSIDE with the same technology [24, 42, 48, 49] and offer them as nutraceuticals alongside Active Botanicals Research PCTC‐based and verbacosid‐containing product ACETOS 10P, which has been approved as novel food (see also Section 4) in Europe [25, 44, 45].

### 2.2 Principles and technologies

#### 2.2.1 Three main cell culture types

**FIGURE 1:**
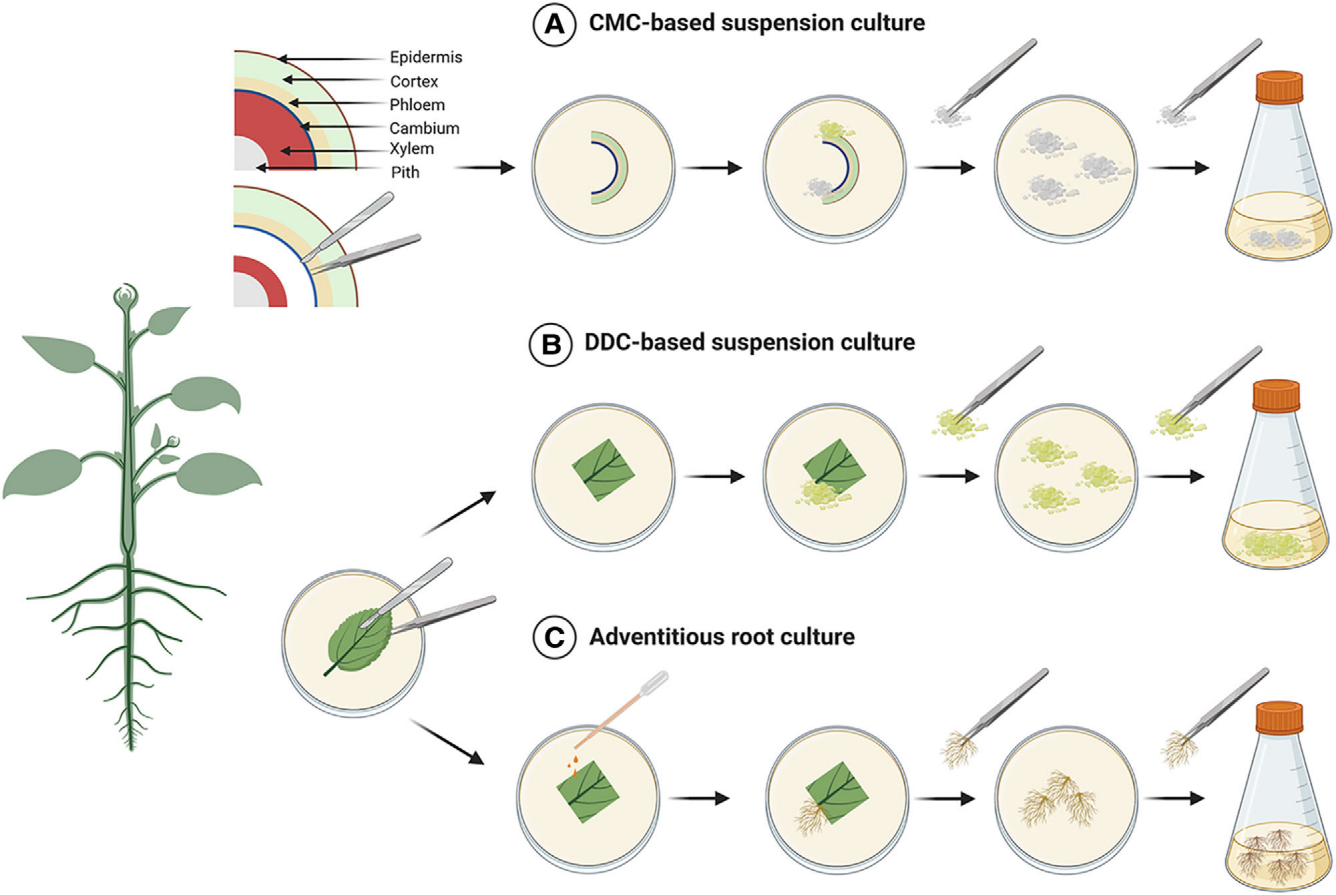
Schematic representation of the procedure for the establishment of PCTCs: (A) CMC‐based suspension culture, (B) DDC‐based suspension culture and (C) Adventitious root culture. Created with Biorender.com

The majority of the products shown in Table 1 are based on plant suspension cells (dedifferentiated cells, DDCs) grown from callus cultures (Figure 1B). Callus is wound tissue induced through injury using a sterile scalpel, following a three‐stage surface sterilization [50, 51] of the mother plant segment containing the target substance(s) in the highest quality and quantity. For this purpose, the sterilized and injured parts of plants are incubated at temperatures between 23°C and 27°C in the dark or under light on agar plates with solid culture media [52, 53]. This is followed by mass propagation of the callus to obtain sufficient biomass for initiating a suspension culture using shaking flasks. Here it is important to work with a friable callus [54]. An efficient production process in the bioreactor requires a homogeneous, well‐growing and productive suspension culture, which sequentially requires a homogenization procedure [55] for the suspension culture, performed over several weeks. DDC‐based suspension cells have doubling times of between 0.6 and 5 days [52] and typically grow as aggregates [56]. Their secondary metabolites, usually formed intracellularly, are present in concentrations between 0.025 and 5 g L^‐1^ [57, 58] and may decrease with increasing passage number [59].

Cambium meristematic cell (CMC)‐derived suspension cells (Figure 1A), which represent true plant stem cells, grow as single cells [60] facilitating their long‐term storage. They are embedded in meristems located at the tips of shoots, as well as roots, or are contained inside the vascular system and can grow faster as well as reach higher product concentrations than DDC‐based suspension cells [61]. The technology for the generation of CMCs is still in its infancy and has been reported for *Taxus cuspidata* [38], *Catharanthus roseus* [62, 63], *Ginko biloba* and *Solanum lycopersicum* [64], *Tripterygium wilfordii* [65] and *Ocimum basilicum* (Mehring A, University of Kaiserslautern, personal communication, 2019). Unhwa Corporation having secured the world`s first patent technology in plant stem cell isolation is the leading company in this field. The first step in CMC establishment is surface sterilization of the cambium after removal of the pith and xylem. To induce CMC growth, the sterilized explants are transferred into an osmotic agent for 16 to 24 h. Subsequently, the explants are incubated for up to 30 days on a solid growth medium in Petri dishes [57, 66]. Afterwards, the propagated CMCs are sub‐cultivated in Petri dishes with fresh growth medium. If sufficient CMCs are available (100 g L^–1^ fresh weight), shake flask cultures may be prepared and cultivated under standard conditions. No homogenization procedure is necessary.

In order to generate an adventitious root culture (Figure 1C), the callus must first be induced and a maintenance culture in Petri dishes established, as with the DDC‐based suspension culture [67]. Afterwards, root induction is carried out with a solid culture medium that supports root formation. The roots are first propagated in Petri dishes and then in shaking flasks, in order to generate a maintenance culture, the latter also serving in the inoculum production for bioreactor cultivation.

As mentioned in the introduction, no genetically modified cultures have been used for the production of PCTC‐based commercial food products or ingredients. While the potential of genetically modified PCTCs in terms of product titer increases is undisputed, challenges primarily concerning the acceptance of such products in the European market persist. For an overview of methods commonly used to produce genetically modified PCTCs, the interested reader is referred to the reviews by Kowalczyk et al. (2020) and Nielsen et al. (2019) [68, 69].

#### 2.2.2 Bioreactor cultivation

There are two dominant bioreactor types which have become established in commercial production processes for the products in Table 1, depending on the PCTC type used. While for suspension cultures stirred stainless steel bioreactors are preferred, root cultures are cultivated in modified bubble column bioreactors, with maximum working volumes in the m^3^ range [70]. Due to the morphology of the suspension cells, their limitation‐free cultivation on a large scale is easier than those of the adventitious root cultures. However, a possible challenge regarding the cultivation in stirred bioreactors is the strong increase in the viscosity of the culture broth when using plant suspension cultures which propagate very well (maximum biomass between 10 and 18 g dry weight L^–1^ or 200 and 350 g fresh weight L^–1^) [71, 72]. For such applications, it is recommended to equip the bioreactor with impellers close to bioreactor wall [73]. Typically, the vessel of a stirred production bioreactor for plant suspension cultures has a height‐to‐diameter ratio of 2:1 or 3:1, is equipped with one or more Rushton, marine or pitched blade impellers or combinations thereof, baffles and a dynamic seal [55]. Another critical point is foam formation due to polysaccharides or media ingredients secreted during cell growth [74]. This leads to the wall growth phenomenon, which is considerably more pronounced in bubble column bioreactors, due to their height‐to‐diameter ratio of 6:1 or 14:1.

Paek et al. (2005) succeeded in foam reduction by modifying a bubble column bioreactor and designing the balloon‐type bubble bioreactor [29]. This bioreactor type has also been recommended for the mass propagation of tissue cultures [46, 75–77], including adventive root cultures, with the largest balloon type bubble bioreactors (1 and 10 m^3^) used by CBN Biotech for the commercial production of ginseng saponins [78].

**FIGURE 2:**
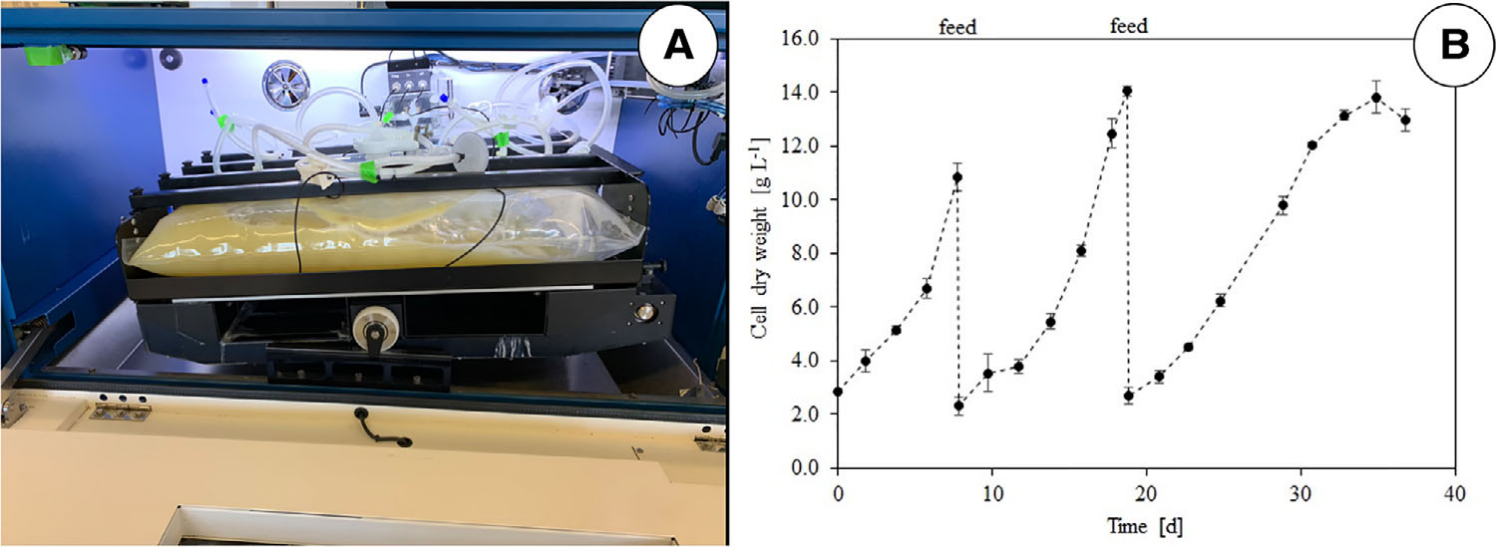
Propagation of *V. vinifera* DDCs in a CELL‐tainer bioreactor system (A) CELL‐tainer containing a 20 L bag. (B) Typical growth curve of the *V. vinifera* suspension cells

The use of single‐use bioreactors [79, 80, 81] for the production of PCTC‐based food ingredients is only beneficial for research and development due to size limitations and the high prices of the pharma‐grade plastic vessels or bags. Due to their homogeneous energy dissipation, wave‐mixed bioreactors [82], such as the BIOSTAT RM (Sartorius), the Wave Bioreactor (Cytiva, formerly GE Healthcare), the HyPerforma Rocker Bioreactor (ThermoFisher) or the CELL‐tainer (Celltainer Biotech) are often used. Figure 2A depicts the CELL‐tainer CT20, in which suspension cells of *Vitis vinifera* were grown. The biomass generated within 38 days (Figure 2B) is comparable to that from a BIOSTAT RM 20/50 operated with a 20 L bag (unpublished data).

Specialists of the Technical Research Center of Finland Ltd. (VTT) developed the first prototype of a 3D printed bioreactor for the cultivation of PCTCs in a domestic kitchen. This aptly named “Home Bioreactor” represents a completely new approach based on a modified bubble column and can be compared to a Nespresso machine [83, 84]. The user inserts a capsule containing PCTC, adds water and turns on the bioreactor. During cultivation, the bioreactor maintains optimal growth conditions until the biomass is harvested and processed. Approximately 500 g of biomass (fresh weight) can be generated within one week and further processed (e.g. muesli) in this manner. The “Home Bioreactor”, however, remains to be made commercially available.

#### 2.2.3 Elicitation for the product titer increase

Regardless of the bioreactor and PCTC type, elicitation approaches have proven to be most effective in the production processes shown in Table 1. For example, product concentrations of secondary metabolites in processes with elicited PCTCs could be increased 55‐fold [85, 86] and in some cases even product secretion [87] could be achieved. Elicitation is a non‐transgenic technique that stimulates secondary metabolite production through physical cues or by adding trace amounts of chemical compounds, called elicitors [88]. The elicitors can either be classified according their origin, as exogenous or endogenous, or on the basis of their nature, as biotic or abiotic [89, 90]. Exogenous elicitors are chemicals originating from outside the target cell, such as fatty acids, polysaccharides, peptides and enzymes, whereas endogenous elicitors include substances such as galacturonide or hepta‐β‐glucosides, which are synthesized inside the target cell by induction of intracellular biotic or abiotic signals [88]. Biotic elicitors are of biological origin, either derived from the plant itself or from a bacterial, fungal or viral pathogen source [88, 91]. Abiotic elicitors cover physical factors (e.g. high pH‐value, temperature shifts, osmolarity, oxidative stress and light) and chemical compounds, such as inorganic salts [91]. Physical elicitation is less investigated, more difficult to monitor and more seldom applied when compared to chemical elicitation.

Since the elicitation process is generally complex and the metabolic pathways not always fully understood, many factors and cultivation conditions may affect the impact of elicitors on the synthesis of secondary metabolites. A popular abiotic elicitor is methyl jasmonate [92] often used in concentrations between 0.5%–10%. By its addition growth and production phase of the PCTC are decoupled [93, 94]. With regard to efficient elicitation procedures, it is important to take into account the complex and time‐consuming preliminary tests required to select the suitable elicitor(s) and to determine the optimum addition time, dosage and exposure time of the elicitor [95].

If an ingredient for the food industry or food itself is to be produced with PCTCs, it is vital that not only the selected elicitor is food grade, but also the other ingredients of the culture medium. Synthetic phytohormones are commonly used as pesticides in agriculture, their usage is therefore regulated (see also Section 4) by the EU pesticide database in Europe and toxicological assessments of intracellular phytohormone accumulation are mandatory [96] if used in PCTC cell culture medium. A study by Häkkinen et al. (2020) evaluated the intracellular accumulation of such phytohormones in arctic bramble and birch cell suspension cultures (DDC‐derived). While they were able to detect free 2.4‐dichlorophenoxyacetic acid (2.4‐D) (0.33–0.83 μg g^–1^ dry weight), the values were below the reported median lethal dose levels measured in rats and mice (320–1000 μg g^‐1^). To circumvent these regulatory hurdles the replacement of synthetic phytohormones, such as 2.4‐D, kinetin and 1‐naphthalenacetic acid, with natural versions such as indole‐3‐acetic acid, casein, yeast extract, zeatin or coconut water [97] can be considered. A completely different approach is the use of hairy root cultures, as these can be cultivated without phytohormones [98]; however, these have yet to be used for the commercial production of food or food ingredients.

## 3 LATEST DEVELOPMENTS

### 3.1 Three new PCTC extracts with potential for human nutrition

Researchers at the Zurich University of Applied Sciences (ZHAW) have succeeded in producing a model chocolate using a DDC‐based *Theobroma cacao* suspension culture propagated in a wave‐mixed bioreactor. The main steps towards establishing mass propagation of the cell culture have already been described by Eibl et al (2018) [99]. The same applies to the production of cell culture chocolate. The sensory analysis by trained chocolate testers showed that the cell culture chocolate, with an intense fruity and sour aroma (comparable to citrus and red berry flavors), presented a promising sensory profile. Chemical analyses showed that the biomass contained both volatile and non‐volatile flavor compounds at a total polyphenol content of 6.69 g kg^–1^. However, the aroma profile of chocolate produced with biomass from the wave‐mixed bioreactor differed from those recently produced in stirred bioreactors [100], with an increase in bitterness observed for the “stirred bioreactor chocolate”. Biomass production in the stirred reactor and wave‐mixed bioreactor were comparable with the harvest of approximately 0.23 kg L^‐1^ biomass (fresh weight) after 16 days. Subsequent research work is now focusing on the possibility of transferring the process from a wave‐mixed bioreactor to a scalable stirred bioreactor and increasing process efficiency.

Another interesting approach to the use PCTC‐based products was described by the researchers of the already mentioned VVT (Section 2.2.2) and is focused on the use of berry suspension cells as food. Nordlund et al. (2017) studied the nutritional properties of DDC‐based plant cell suspension cultures of *Rubus chamaemorus* L., *Vaccinium vitis‐idaea* L. and *Rubus saxatilis* L. [101], also investigating the carbohydrate, lipid and protein composition, in vitro protein digestibility and sensory properties. The results confirm the potential use of the plant suspension cells as a source of food itself for the first time. A fresh odor, as well as flavor and a favorable composition (21%–37% dietary fiber, 0.3%–1% starch, 18%–33% sugars, satisfactory lipid quality, 14%–19% proteins, balanced amino acid profile) of the cell culture biomass were achieved. Furthermore, it was shown that it was possible to mass propagate *Rubus chamaemorus* L suspension cells up to pilot scale (300 L working volumes, stirred bioreactor, feeding) [102]. Interestingly, flavanols, to which beneficial health effects are attributed [103], are atypical for *Rubus* fruits cultivated in the field or collected in nature.

Finally, Bianconi et al. (2020) demonstrated the industrial potential of a red carrot cell line extract (R4G extract) for food application (colorant and health food ingredient). It is based on DDC‐derived suspension cells (dark culture) of *Daucus carota*, which were grown in Gamborg's B5 medium on shake flask and 50 L bioreactor (30 L working volume) scale [104]. The establishment of the production cell line was described by Ceoldo et al. (2005) and Ceoldo et al. (2009) [105, 106]. The R4G extract is characterized by large quantities of anthocyanins, which were higher and more stable than those found in natural red carrot extract, while the metabolic profiles of both extracts were comparable. Furthermore, a noticeable increase of anthocyanin content was achieved by increasing the sucrose level in the culture medium from 25  to 40 g L^–1^. The antioxidant and anti‐inflammatory activities of the R4G extract were confirmed in vivo using mice.

### 3.2 PCTC‐derived citrus oil ingredients

**FIGURE 3:**
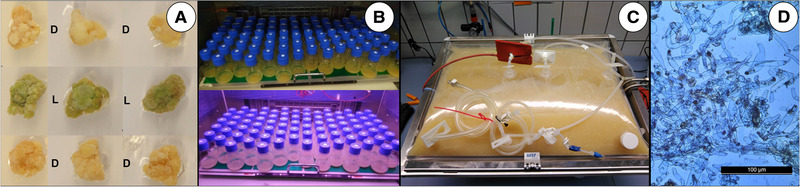
Citrus fruit derived PCTCs grown in the dark (D) or under light (L) regime (16 h/8 h): (A) Callus cell lines (B) Suspension cell lines (C) Suspension cell culture in a 20 L wave‐mixed bioreactor (D) Light microscopy image of citrus fruit vesicles in suspension culture

Citrus, one of the most important crops worldwide, is essential for both the beverage and flavor industry [107, 108, 109] amongst others. However, climate change, dwindling potable water supplies, soil salinity and plant diseases (e.g. citrus greening disease) has led to supply issues concerning citrus fruit and their products. As these threats are only expected to grow in the near future, callus and suspension cultures of numerous citrus varieties have been established and the PCTCs capabilities of producing typical citrus oil ingredients have been investigated (Figure 3).

Analysis showed that even in the callus tissue various known citrus volatiles, in the range of several hundred mg/kg dry weight, could already be found. To further increase the production of citrus volatiles, a precursor [110, 111, 112] feeding approach was used. This concept is based on the idea that a natural derived, less expensive substance (intermediate) can be added, in order to induce and increase the production of the compound(s) of interest [18, 113].

To achieve this, a suspension culture (Figure 3B) originating from the flavedo of a citrus fruit was used. The doubling time of the suspension cells (batch culture, 26°C, 120 rpm) was 3.9 days. A two‐step process (10 days growth and 14 days production) was developed on a shaking flask scale (100 mL working volume). After adding the precursor (sesquiterpene), a balsamic fruity scent of orange flower accompanied by a strong citrus taste was produced. Following extraction of the biomass, 37 volatile compounds from various organic classes (aldehydes, alcohols, ethers, furans and other) were detected by gas chromatography‐mass spectrometry analysis of the headspace and solvent. To enable quantitative studies, the process was scaled up to a wave‐mixed BIOSTAT RM 20/50 (Figure 3C), with a working volume of 10 L. Upon completion of the cultivation, 2 kg of biomass fresh weight and 4.5 L of liquid were harvested. Both the biomass and culture supernatant carried the balsamic fruity orange flower like scent, while the taste of the liquid was described as that of white grapefruit by a flavorist of Givaudan. Approximately, 0.6% of the terpenic compound, responsible for this characteristic scent and taste, was detected in the biomass dry weight, twofold of that which can be found naturally occurring in the fruit. However, in order to improve the efficiency and commercial viability of the developed method, further process scale‐up to a stirred stainless‐steel system in the m^3^ range is necebssary.

## 4 REGULATORY ISSUES

The progress in the PCTC technology over the last two decades demonstrates that this technology has great potential as an alternative production method in the food sector. As already written, only a handful of PCTC based products have been commercialized. Besides the challenges on process efficiency, the complex regulatory landscape is another limiting factor for their commercialization. As countries have their own regulatory frameworks, the definitions as well as approval processes may vary, which may result in an increase in time, as well in costs to get a product to market.

Already, the definition of a novel food/food ingredient varies broadly. In Europe a food is considered as novel and will fall in the scope of the EU Regulation 2015/2283 on novel food, if it has not been used to a significant degree for food consumption in the EU before May 15, 1997. Food additives (e.g. colorants) and flavourings are not in the scope of this legislation and are covered by their own legislative framework with separate authorisation procedures. In the USA all substances added intentionally to food are considered as “food additives” and require pre‐market approval by the FDA, unless the substance is generally recognized as safe (GRAS) through scientific procedures, through safe history of use in food (dating to before 1958), or it meets one of the other exclusions from the food additive definition in section 201(s) of the Federal Food, Drug and Cosmetic Act.

Although the path to approval of different categories of food additives varies from jurisdiction to jurisdiction, there are many commonalities in terms of the data requirements and considerations for assessment regarding the safety of use of food additives, flavouring or novel food substances, including the use of positive lists of approved substances, pre‐market approval, as well as separation between science and policy decisions. All the different approaches do have a main purpose in common, which is to ensure the safety of the consumed food.

The safety of food regarding traditional use is usually accepted on the basis of its history of safe use. Within a safety assessment, traditional foods/food ingredients are used as reference points, whereas this approach is based on the concept of substantial equivalence of the traditional food/food ingredients versus the novel food/food ingredient under assessment. However, this approach has its limitation in the space of PCTCs, as many PCTCs do not necessarily deliver the same product profile as the whole plant part does. Furthermore, it is mostly not sufficient to take only the source materials and its composition into account, as all characteristics of the product as well as the production process needs to be assessed.

Even the EU Regulation 2015/2283 on novel food clearly defines that food consisting of, isolated from, or produced from a PCTC is considered as one of the novel food categories listed in the EU and requires pre‐market authorisation, which includes a safety assessment performed by the European Food Safety Authority (EFSA). For proper characterisation of the novel food, EFSA has provided guidance [114] in which the specific data requirements in relation to PCTCs are described.

Currently the PCTC extract from *Ajuga reptans, Lippia citriodora* and *Echinacea augustifolia* suspension cells (Table 1, Section 2.1) had been authorised as novel food for the use in food supplements under EU Regulation 2015/2283 and therefore imparted in the Union List of authorised novel foods (Commission Implementing Regulation EU 2017/2470). So far, no authorizations have been granted in Europe for food, like e.g. anthocyanins under the food additive legislation.

Another important factor for successful commercialization is the acceptance of the consumer. Consumers can sometimes be hesitant in accepting a novel food technology, even if it has already been perceived as safe by the experts. Most consumers viewed food manufactured with a minimum of processing with more positive attributes than highly processed foods or food manufactured using new technologies. As some authorisations might include specific labelling requirements as, for example in the case of *Lippia citriodora* cell culture extract, for which the designated labelling of the foodstuffs containing shall be ‘dried extract of *Lippia citriodora* from cell cultures HTN®Vb’, consumer acceptance can be a challenging factor.

## 5 CONCLUDING REMARKS

The potential of PCTCs for the sustainable and controlled production of food ingredients is undisputed. Today, the number of PCTC‐based products in the food sector, representing colorants and substances stimulating the immune system or having health effects, is still limited. However, this could change in the near future through climate change, loss of arable land and potable water designated for food production, and plant diseases. Furthermore, smooth regulatory approval and consumer acceptance of new PCTC‐based products will play an important role in their success, with single‐use bioreactors, such as wave‐mixed systems, supporting and accelerating process development. In addition to elicitor and/or precursor feeding, experimental design may improve process efficiency by increasing biomass yield more than twofold and reducing the cost of goods by more than half, as demonstrated by Rasche et al. (2016) [113]. This could pave the way for increased use of PCTCs in the commercial production of food [96] and food ingredients.

## CONFLICT OF INTEREST

The authors have declared no conflicts of interest.

## Data Availability

The data that support the findings of this study are available from the corresponding author upon reasonable request.
